# Comparison of glaucoma patients referred by glaucoma screening versus referral from primary eye clinic

**DOI:** 10.1371/journal.pone.0210582

**Published:** 2019-01-10

**Authors:** Yong Ju Song, Yong Woo Kim, Ki Ho Park, Young Kook Kim, Hyuk Jin Choi, Jin Wook Jeoung

**Affiliations:** 1 Department of Ophthalmology, Chosun University Hospital, Chosun University College of Medicine, Gwangju, Korea; 2 Department of Ophthalmology, Seoul National University Hospital, Seoul National University College of Medicine, Seoul, Korea; 3 Healthcare System Gangnam Center, Seoul National University Hospital, Seoul, Korea; Bascom Palmer Eye Institute, UNITED STATES

## Abstract

**Purpose:**

To investigate whether mass glaucoma screening relative to opportunistic case finding at a primary eye clinic is helpful for early detection of glaucoma.

**Methods:**

Subjects referred by glaucoma screening (by non-contact tonometry and non-mydriatic fundus photography; group A, *n* = 220) and from a primary eye clinic (group B, *n* = 327) were retrospectively recruited. The positive predictive value (PPV) for glaucoma and the rate of glaucoma awareness were compared. Also, for the newly diagnosed glaucoma (‘definite glaucoma’) patients, the demographics and structural and functional severities of glaucoma were compared.

**Results:**

The PPV for definite glaucoma was 25.5% for group A and 52.4% for group B. The rate of false-positive for ‘glaucoma referral to tertiary hospital’ was significantly higher for group A than for group B (38.6 vs. 18.3%, P < 0.001). Among the definite-glaucoma patients (group A: *n* = 56; group B: *n* = 182), the proportion of glaucoma awareness was significantly higher in group B (69.2%) than in group A (8.9%, P < 0.001). The mean deviation (MD) of visual field (VF) was significantly higher in group A than in group B (–3.08 ± 3.99 vs. –6.70 ± 7.29 dB, *P*_adjusted_ = 0.040), and the inferior and inferotemporal ganglion cell-inner plexiform layer (GCIPL) thicknesses tended to be greater in group A than in group B, with marginal significance (*P*_adjusted_ < 0.10).

**Conclusions:**

Glaucoma screening can be helpful for early detection of glaucoma. However, improvement of the screening strategy is needed in order to enhance its specificity for glaucoma.

## Introduction

Glaucoma affects over 60 million patients worldwide[1]; its reported global prevalence is 3.54% among those aged 40 to 80.[2] It is notorious for progressive visual field defect and blindness where left untreated or treated inappropriately or inadequately.[3] IOP-lowering treatment, by medication or surgery, has only added evidence for the necessity of preventing glaucoma progression.[4] As most early cases are painless and asymptomatic, glaucoma often is diagnosed late, which is a significant public health problem. For these reasons, glaucoma has been a target of public screening programs for early diagnosis and appropriate management.

Against expectations, the US Preventive Services Task Force (USPSTF) concluded that the evidence justifying glaucoma screening for adults who do not have signs or symptoms of glaucoma or other vision problems is not sufficient.[5] However, this recommendation is debatable, as it is predicated only on glaucoma screening provided by a primary care professional (not eye specialists such as ophthalmologists or optometrists). The American Academy of Ophthalmology (AAO)’s *Primary Open-Angle Glaucoma Preferred Practice Pattern((R)) Guidelines* indicate that glaucoma screening can be useful for high-risk populations such as the elderly, African Americans and Hispanic Americans, and those with a family history of glaucoma.[6]

Given the especially high prevalence of normal-tension glaucoma (NTG) in East Asian countries (including Korea), glaucoma screening in this region might be more efficacious than in Western countries[7], particularly considering the region’s high prevalence of myopia [8], a well-known risk factor for glaucoma development[9].

In this regard, the present study compared the general demographic characteristics and structural and functional glaucoma severities of patients referred by glaucoma screening (by non-contact tonometry and non-mydriatic fundus photography) with those of patients referred from primary eye clinics. It concluded, based on the comparative results, that glaucoma screening in regions where NTG and/or myopia prevalence is high can be helpful for early detection of glaucoma.

## Materials and methods

### Subjects

The present study included subjects from the Gangnam Eye Study, an ongoing cohort study conducted by the Gangnam Healthcare Center of Seoul National University Hospital. The detailed information on this cohort has been published elsewhere.[10] The study population comprises subjects who had participated in a glaucoma screening program at the Gangnam Healthcare Center of Seoul National University Hospital (SNUH). In the current study, 221 subjects who had been referred, post-screening, to SNUH Glaucoma Outpatient Clinic between January 2013 and December 2014 were enrolled. Another 326 subjects who had been referred from a primary eye clinic during the same period were retrospectively recruited. Subjects with a history of any retinal disease (e.g., diabetic retinopathy, retinal vein occlusion, age-related macular degeneration) or a history of intraocular surgery (except uncomplicated cataract surgery) that could affect the visual field (VF) were excluded. The present study was approved by the Seoul National University Hospital (SNUH) Institutional Review Board (IRB) (IRB No. 1807-098-960) and followed the tenets of the Declaration of Helsinki (1964).

### Glaucoma-screening program

The glaucoma-screening examination is comprised of IOP measurement by non-contact tonometry (model CT10; Topcon Inc., Tokyo, Japan) and fundus photography by a non-mydriatic fundus camera (model EOD D060; Canon Inc., Utsunomiya, Japan). The fundus photographs were evaluated by an experienced ophthalmologist (H.J.C.) for suspicious findings such as glaucomatous optic nerve head (ONH) change or retinal nerve fiber layer (RNFL) defect. The detailed criteria for referral for definitive glaucoma examination were as follows.

1. Suspected glaucomatous optic neuropathy: vertical cup-to-disc ratio (VCDR) ≥ 0.6, VCDR difference ≥ 0.2 between the eyes, minimal neural rim width < 0.1 times the disc diameter, or disc hemorrhage.
2. Suspected RNFL defect: width greater than that of a major retinal vessel when measured at the disc edge, diverging in an arcuate or wedge shape.
3. Baseline IOP > 21 mmHg.

### Glaucoma referral from primary eye clinic

Subjects who had visited a primary eye clinic and been diagnosed as glaucoma suspect were referred to SNUH Glaucoma Outpatient Clinic for further evaluation. All of the subjects had undergone slit-lamp examination, IOP measurement, and fundus examination.

### Definitive glaucoma examination

All of the referred subjects underwent a complete ophthalmic examination including best-corrected visual acuity (BCVA), refraction, Goldmann applanation tonometry, gonioscopy, stereo optic-disc photography, red-free fundus photography (CF-60Uvi; Canon Inc., Utsunomiya, Japan), SD-OCT (Cirrus; Carl Zeiss Meditec, Inc., Dublin, CA, USA) and standard automated perimetry (Humphrey C 24–2 SITA-Standard visual field; Carl Zeiss Meditec). Also, the central corneal thickness (CCT) (Pocket II; Quantel Medical, Clermont-Ferrand, France) and axial length (AXL) (AXIS-II Ultrasonic Biometer; Quantel Medical S.A., Bozeman, MT, USA) were measured.

Open-angle glaucoma (OAG) was defined as eyes manifesting glaucomatous optic disc changes such as focal notching and thinning, RNFL defects on disc stereo-photography and red-free fundus photography, glaucomatous VF defect, and an open-angle confirmed by gonioscopic examination. Glaucomatous VF defect was defined as (1) glaucoma hemifield test values outside the normal limits or (2) three or more abnormal points with a probability of being normal of *P* < 5%, of which at least one point had a pattern deviation of *P* < 1%, or (3) a pattern standard deviation (PSD) of *P* < 5%. The VF defects were confirmed on two consecutive reliable tests (fixation loss rate ≤ 20%, false-positive and false-negative error rates ≤ 25%).

Glaucoma suspect was defined as typical glaucomatous optic disc changes with open-angle but with the absence of compatible VF defect. Eyes with no abnormalities on disc stereo-photography, red-free fundus photography, or standard automated perimetry were regarded as normal. NTG was defined as OAG with IOP < 21 mmHg.

Diagnosis of glaucoma was performed by an experienced ophthalmologist (J.W.J). If both eyes were eligible, one eye was randomly selected as the study eye.

### Data analyses

The positive predictive values (PPV) of referral by glaucoma screening and referral from a primary eye clinic were calculated for definite glaucoma and for glaucoma suspect. The proportion of glaucoma awareness, defined as the ratio of the number of definite-glaucoma patients with a history of IOP-lowering treatment to the total number of definite-glaucoma patients, were calculated and compared between the groups. To investigate how specific the two diagnostic strategies (glaucoma screening vs. referral from primary eye clinic) are for glaucoma diagnosis, the false-positive rate for ‘glaucoma referral to a tertiary hospital (SNUH)’ was measured and compared between the groups. To compare the glaucoma characteristics identified through each strategy, the newly diagnosed glaucoma patients (51 patients by glaucoma screening and 56 from a primary eye clinic) were evaluated and compared for their demographics as well as structural (OCT-measured RNFL and macular ganglion cell-inner plexiform layer [GCIPL] thicknesses) and functional (HVF indices) characteristics. The statistical analyses were performed using SPSS 21.0 (SPSS, Inc., Chicago, IL, USA). Student t-test was used for comparison of continuous variables, and Pearson chi-square test for categorical variables. The false discovery rate (FDR) was controlled for using the Benjamini-Hochberg method.[11] Except where stated otherwise, the data are presented as mean ± standard deviations, and the level of statistical significance was *P* < 0.05.

## Results

### Subject demographics

A total of 547 eyes of 547 patients were included in this study. Among them, 220 patients were referred by glaucoma screening (by non-contact tonometry and non-mydriatic fundus photography; group A) and 327 were referred from a primary eye clinic (group B). Group B (mean age: 55.7 ± 14.8 years) was significantly older than group A (mean age: 51.7 ± 11.4; adjusted *P* < 0.001), and more female than male (55 vs. 43%, adjusted *P* = 0.025). There were no significant differences in IOP, refraction or AXL between the two groups (**Table 1**), but the CCT was significantly thinner in group B (544.2 ± 36.5 μm vs. 535.3 ± 40.3, adjusted *P* = 0.028).

**Table 1:** Subjects’ demographics.

Variable	Group A (*n* = 220)	Group B (*n* = 327)	Adjusted *P*-value
**Age, yrs**	**51.7 ± 11.4**	**55.7 ± 14.8**	<0.001*
**Gender, female, *n* (%)**	**94 (43%)**	**178 (55%)**	**0.025**†
FHx of glaucoma, *n* (%)	14 (6%)	32 (10%)	0.22†
IOP, mmHg	14.0 ± 2.5	14.8 ± 5.7	0.05*
Spherical equivalence, *D*	–1.76 ± 3.05	–1.95 ± 3.32	0.64*
Non-myopia	75 (34%)	121 (37%)	0.56†
Mild-to-moderate myopia	122 (55%)	170 (52%)	
High myopia	23 (11%)	36 (11%)	
Axial length, mm	24.36 ± 2.24	24.42 ± 2.14	0.78*
**CCT, μm**	**544.2 ± 36.5**	**535.3 ± 40.3**	0.028*

Group A: subjects from glaucoma screening test, Group B: subjects referred from primary eye clinic

FHx = Family history; IOP = intraocular pressure; D = diopters; CCT = central corneal thickness

Non-myopia: SE ≥ 0 D, Mild-to-moderate myopia: –6 D < SE < 0 D, High myopia: SE ≤ –6 D

* Comparison of groups by Student-t test

† Comparison of groups by Pearson chi-square test

P-values adjusted by Benjamini-Hochberg method to compensate for multiple comparison

### Positive predictive value and glaucoma awareness

**Fig 1:**
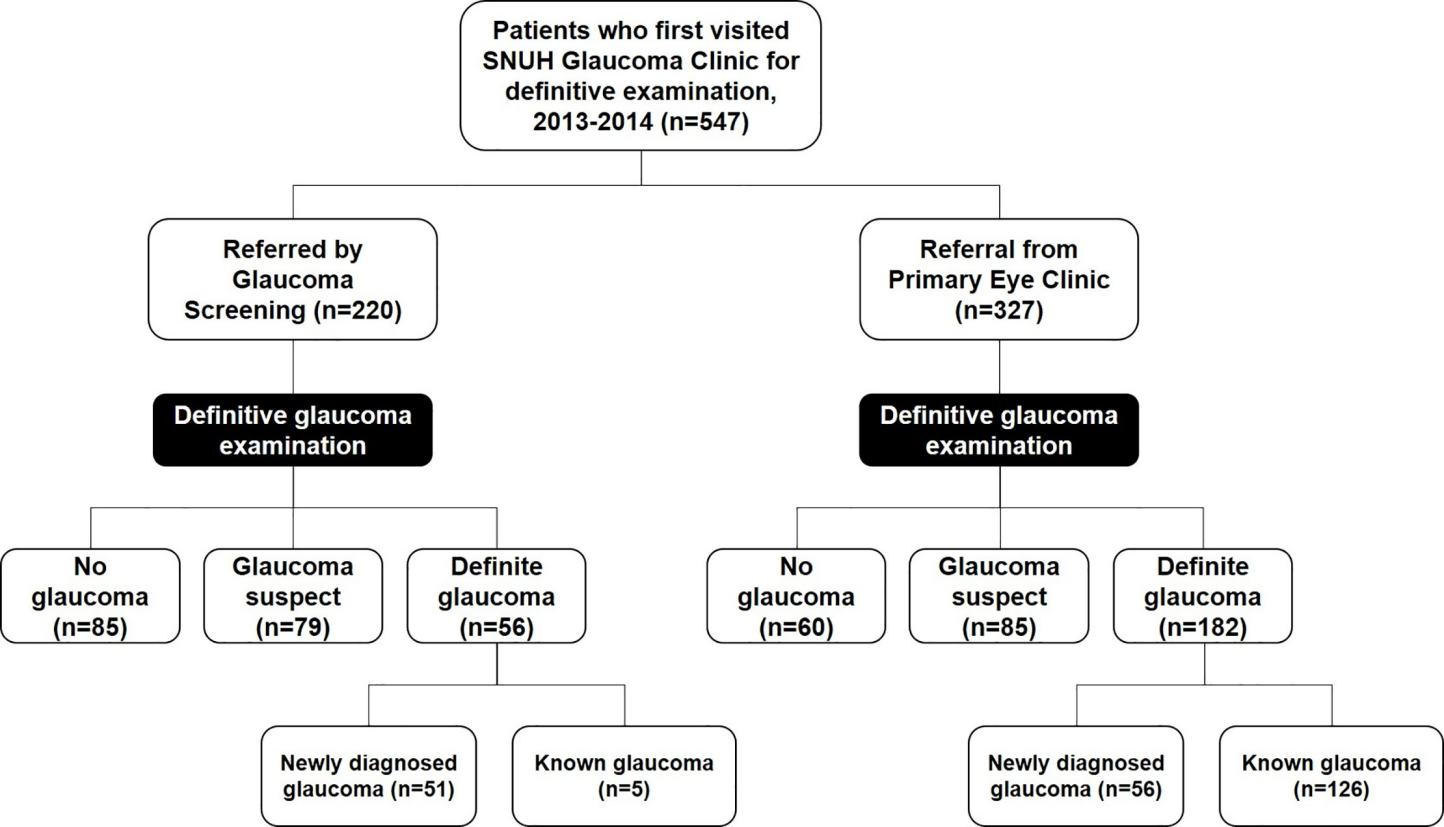
Flow diagram of participant progress.

In group A, 56 patients were diagnosed with definite glaucoma, 79 were classified as glaucoma suspect, and 85 were revealed to have normal eyes. In group B, 182 patients were diagnosed with definite glaucoma, 85 were classified as glaucoma suspect, and 80 were revealed to have normal eyes (**Fig 1**).

The PPV for definite glaucoma was 25.5% for group A and 52.4% for group B. The PPV for glaucoma or glaucoma suspect was 61.4% for group A and 76.9% for group B. The false-positive rate for ‘glaucoma referral to a tertiary hospital (SNUH)’ was significantly higher in group A (38.6%) than in group B (18.3%, *P* < 0.001).

Among the definite-glaucoma patients (group A: *n* = 56; group B: *n* = 182), the proportion of glaucoma awareness was significantly higher in group B (*n* = 126, 69.2%) than in group A (*n* = 5, 8.9%) (*P* < 0.001).

### Comparison of glaucoma characteristics of newly diagnosed glaucoma patients

Additionally, the glaucoma characteristics of the newly diagnosed glaucoma patients in groups A (*n* = 51) and B (*n* = 56) were compared. There were no significant differences in age, gender, refraction, AXL, CCT, or average and vertical C/D between the two groups (**Table 2**). However, the IOP was significantly lower in group A (14.4 ± 3.1 mmHg) than in group B (17.3 ± 7.3 mmHg, adjusted *P* = 0.040). The proportion of NTG was significantly higher in group A (*n* = 47, 92.2%) than in group B (*n* = 38, 67.9%) (*P* = 0.004).

**Table 2:** Inter-group comparison of newly diagnosed glaucoma patients.

Variable	Group A (*n* = 51)	Group B (*n* = 56)	Adjusted *P*-value
Age, yrs	52.5 ± 11.1	52.9 ± 14.8	0.89*
Gender, female, *n* (%)	20 (39%)	26 (47%)	0.63^†^
**IOP, mmHg**	**14.4 ± 3.1**	**17.3 ± 7.3**	0.040*
Spherical equivalence, *D*	–2.74 ± 3.60	–2.61 ± 3.25	0.89*
Axial length, mm	24.32 ± 3.70	24.95 ± 1.59	0.47*
CCT, μm	540.6 ± 46.8	538.6 ± 45.4	0.89*
Average C/D	0.69 ± 0.12	0.74 ± 0.10	0.14*
Vertical C/D	0.70 ± 0.12	0.74 ± 0.10	0.21*
**MD of VF, dB**	**–3.08 ± 3.99**	**–6.70 ± 7.29**	0.040*
PSD of VF, dB	5.01 ± 4.22	6.14 ± 4.20	0.40*

Group A: subjects from glaucoma screening test, Group B: subjects referred from primary eye clinic

IOP = intraocular pressure; D = diopters; CCT = central corneal thickness; C/D = cup-to-disc ratio; MD = mean deviation; VF = visual field; PSD = pattern standard deviation

* Comparison of groups by Student-t test

† Comparison of groups by Pearson chi-square test

P-values adjusted by Benjamini-Hochberg method to compensate for multiple comparison

The mean deviation (MD) of VF was significantly higher in group A than in group B (–3.08 ± 3.99 vs. –6.70 ± 7.29 dB, adjusted *P* = 0.040), but not for the PSD of VF (5.01 ± 4.22 vs. 6.14 ± 4.20 dB, adjusted *P* = 0.40). In a comparison of the OCT parameters, group A tended to show higher values for RNFL and macular GCIPL thicknesses, but the differences did not reach statistical significance (**Table 3**). The GCIPL thicknesses for the average, minimum, superior, inferotemporal and inferior sectors showed significantly greater values in group A than in group B (all *P*s < 0.05), but the adjustment for multiple comparisons revealed only marginal statistical significance for the inferior and inferotemporal thicknesses (all adjusted *P*s < 0.10).

**Table 3:** Comparison of Optical Coherence Tomography (OCT) parameters of newly diagnosed glaucoma patients.

Variable	Group A (*n* = 51)	Group B (*n* = 56)	Adjusted *P*-value*
**RNFL thickness,** μm			
Average	77.2 ± 10.6	73.1 ± 11.7	0.18
Superior	98.9 ± 17.8	90.6 ± 22.3	0.10
Nasal	61.5 ± 8.6	59.6 ± 9.9	0.36
Inferior	83.8 ± 22.3	76.4 ± 18.2	0.12
Temporal	61.1 ± 12.6	61.3 ± 12.0	0.95
**GCIPL thickness,** μm			
Average	72.4 ± 8.3	68.5 ± 8.8	0.08
Minimal	62.7 ± 11.8	57.2 ± 12.8	0.10
Superotemporal	73.0 ± 8.9	70.7 ± 12.9	0.36
Superior	75.9 ± 9.7	71.5 ± 10.5	0.10
Superonasal	77.0 ± 9.6	74.0 ± 10.2	0.19
Inferonasal	72.1 ± 14.1	70.0 ± 10.2	0.43
Inferior	68.6 ± 10.6	63.3 ± 11.3	0.08
Inferotemporal	66.6 ± 10.7	61.2 ± 11.3	0.08

RNFL = retinal nerve fiber layer; GCIPL = ganglion cell-inner plexiform layer

* Comparison of groups by Student-t test

P-values adjusted by Benjamini-Hochberg method to compensate for multiple comparison

## Discussion

The present study investigated the efficacy of glaucoma screening compared with opportunistic case finding from a primary eye clinic among a Korean population in which NTG and/or myopia prevalence could be expected to be high. The obtained data demonstrated that glaucoma patients identified by glaucoma screening had, relative to those referred from a primary eye clinic, significantly milder VF defect and tended to have greater RNFL and macular GCIPL thicknesses, though the latter differences were not significant after correction for multiple comparisons.

The Korea National Health and Nutrition Examination Survey (KNHNES) 2008–2011 revealed the prevalence of POAG in the Korean population to be 4.7%, which increased with age.[12] Among the participants diagnosed with glaucoma, only 8.0% were aware of the disease. Our data showed a consistent finding: 8.9% awareness among the glaucoma-screening group. As the present study demonstrated that glaucoma eyes referred by glaucoma screening had milder VF defect than glaucoma eyes referred from a primary eye clinic, glaucoma screening can be considered to be helpful for early detection of glaucoma in those unaware of the disease. The present finding is consistent with two previous reports that validated the efficacy of glaucoma screening for visual morbidity by projection or a microsimulation model in African Americans.[13, 14]

The PPV of glaucoma screening was only 25.5% for definite glaucoma and 61.4% when including glaucoma-suspect eyes. The false-positive rate for glaucoma referral was significantly higher in group A (glaucoma screening) than in group B (primary eye clinic). This phenomenon can be explained by following speculations. First, this might be related to glaucoma prevalence and the strategy of glaucoma screening. Because the patients of group B were older than those of group A, it might have included more glaucoma cases, as glaucoma prevalence increases with age. For the purposes of the present study, glaucoma screening included only non-contact tonometry and non-mydriatic fundus photography. By contrast, subjects referred from a primary eye clinic had undergone not only tonometry and fundus examination but also, in some cases, SD-OCT or standard automated perimetry. Glaucoma diagnosis by multimodal tests might improve specificity, but can also increase cost. Evaluating the cost-effectiveness of glaucoma screening certainly is a pertinent and important issue, but was beyond the scope of the present study. Second, the present study defined the ‘definite glaucoma’ as the eyes with definite glaucomatous VF defect, so that the preperimetric cases would have been classified to ‘glaucoma suspect’ group. As the proportion of the glaucoma suspect eyes were significantly higher for group A (35.9%) than group B (26.0%, *P* = 0.017), the higher proportion of preperimetric glaucoma cases in glaucoma screening population (group A) may have lowered the PPV for ‘definite glaucoma’. Considering that the glaucoma patients have changes to the ONH and RNFL prior to VF defect, our findings can be another evidence for the value of glaucoma screening for early detection of glaucoma.

Interestingly, the IOP was significantly lower in group A than in group B when comparing newly diagnosed glaucoma patients. Many healthcare screening programs use IOP as one of the screening tests for glaucoma. However, IOP measurement by itself will miss approximately 50% of patients with glaucoma, due to its low sensitivity and specificity.[15] This effect will be more outstanding in regions where NTG is highly prevalent. In this regard, non-contact tonometry might not be an appropriate tool for glaucoma screening.[16] Instead, non-mydriatic fundus photography is a relatively inexpensive, objective, and non-invasive means of detecting glaucomatous change of the ONH.[17, 18] The present findings demonstrated that mass screening by use of non-mydriatic fundus photography can be more helpful for earlier detection of glaucoma than opportunistic case findings at a primary eye clinic. However, it was not an effective screening method in terms of specificity, as a higher false-positive rate was found there than among the cases of referral from a primary eye clinic. Further improvement in the screening strategy, including a newer technique for diagnosis of glaucoma, is necessary in order to enhance its specificity for glaucoma detection.

The present study has the following shortcomings. First, it did not investigate the issues of cost-effectiveness or quality-of-life improvement for glaucoma patients, which also are important public health concerns. Second, subjects from glaucoma screening are likely to have high socioeconomic, educational status and high interest in health, who are motivated to pay high costs for their health checkup. By contrast, subjects from primary eye clinic are from different areas, and may have diverse interests in their health status. Due to the limitation from retrospective design, it was hard to evaluate and control for these bias, which might have affected on the present data. Third, it did not evaluate the rate of glaucoma progression. Further prospective studies will confirm whether early detection of glaucoma by glaucoma screening is beneficial for prevention of glaucoma progression.

In conclusion, mass glaucoma screening by non-mydriatic fundus photography detected earlier cases of glaucoma compared with opportunistic case findings at a primary eye clinic. Glaucoma screening can be helpful for early detection of the disease, especially in regions where NTG and/or myopia prevalence is high. However, further improvement in the screening strategy is needed in order to enhance its specificity.

## Supporting information

S1 Dataset (XLSX)

## References

[1] Quigley HA, Broman AT. The number of people with glaucoma worldwide in 2010 and 2020. The British journal of ophthalmology. 2006;90(3):262–7. Epub 2006/02/21. 10.1136/bjo.2005.081224 16488940 PMC1856963

[2] Tham YC, Li X, Wong TY, Quigley HA, Aung T, Cheng CY. Global prevalence of glaucoma and projections of glaucoma burden through 2040: a systematic review and meta-analysis. Ophthalmology. 2014;121(11):2081–90. Epub 2014/07/01. 10.1016/j.ophtha.2014.05.013 . 24974815

[3] Quigley HA. Glaucoma. Lancet. 2011;377(9774):1367–77. Epub 2011/04/02. 10.1016/S0140-6736(10)61423-7 . 21453963

[4] Weinreb RN, Aung T, Medeiros FA. The pathophysiology and treatment of glaucoma: a review. JAMA: the journal of the American Medical Association. 2014;311(18):1901–11. Epub 2014/05/16. 10.1001/jama.2014.3192 24825645 PMC4523637

[5] Moyer VA, Force USPST. Screening for glaucoma: U.S. Preventive Services Task Force Recommendation Statement. Annals of internal medicine. 2013;159(7):484–9. Epub 2013/12/11. 10.7326/0003-4819-159-6-201309170-00686 . 24325017

[6] Prum BE Jr., Rosenberg LF, Gedde SJ, Mansberger SL, Stein JD, Moroi SE, et al Primary Open-Angle Glaucoma Preferred Practice Pattern((R)) Guidelines. Ophthalmology. 2016;123(1):P41–P111. Epub 2015/11/20. 10.1016/j.ophtha.2015.10.053 . 26581556

[7] Cho HK, Kee C. Population-based glaucoma prevalence studies in Asians. Survey of ophthalmology. 2014;59(4):434–47. Epub 2014/05/20. 10.1016/j.survophthal.2013.09.003 . 24837853

[8] Morgan IG, French AN, Ashby RS, Guo X, Ding X, He M, et al The epidemics of myopia: Aetiology and prevention. Progress in retinal and eye research. 2018;62:134–49. Epub 2017/09/28. 10.1016/j.preteyeres.2017.09.004 . 28951126

[9] Marcus MW, de Vries MM, Junoy Montolio FG, Jansonius NM. Myopia as a risk factor for open-angle glaucoma: a systematic review and meta-analysis. Ophthalmology. 2011;118(10):1989–94 e2. Epub 2011/06/21. 10.1016/j.ophtha.2011.03.012 . 21684603

[10] Kim YK, Choi HJ, Jeoung JW, Park KH, Kim DM. Five-year incidence of primary open-angle glaucoma and rate of progression in health center-based Korean population: the Gangnam Eye Study. PLoS One. 2014;9(12):e114058 Epub 2014/12/05. 10.1371/journal.pone.0114058 25474589 PMC4256402

[11] Hochberg Y, Benjamini Y. More powerful procedures for multiple significance testing. Stat Med. 1990;9(7):811–8. Epub 1990/07/01. . 2218183 10.1002/sim.4780090710

[12] Kim KE, Kim MJ, Park KH, Jeoung JW, Kim SH, Kim CY, et al Prevalence, Awareness, and Risk Factors of Primary Open-Angle Glaucoma: Korea National Health and Nutrition Examination Survey 2008–2011. Ophthalmology. 2016;123(3):532–41. Epub 2016/01/10. 10.1016/j.ophtha.2015.11.004 . 26746594

[13] Blumberg DM, Vaswani R, Nong E, Al-Aswad L, Cioffi GA. A comparative effectiveness analysis of visual field outcomes after projected glaucoma screening using SD-OCT in African American communities. Invest Ophthalmol Vis Sci. 2014;55(6):3491–500. Epub 2014/05/03. 10.1167/iovs.14-14014 24787570 PMC4073998

[14] Ladapo JA, Kymes SM, Ladapo JA, Nwosu VC, Pasquale LR. Projected clinical outcomes of glaucoma screening in African American individuals. Archives of ophthalmology. 2012;130(3):365–72. Epub 2012/03/14. 10.1001/archopthalmol.2011.1224 . 22411665 PMC8075062

[15] Eddy DM, Sanders LE, Eddy JF. The value of screening for glaucoma with tonometry. Survey of ophthalmology. 1983;28(3):194–205. Epub 1983/11/01. . 6422575 10.1016/0039-6257(83)90097-8

[16] Spaeth GL. Proper outcome measurements regarding glaucoma: the inadequacy of using intraocular pressure alone. European journal of ophthalmology. 1996;6(2):101–5. . 8823579 10.1177/112067219600600201

[17] Detry-Morel M, Zeyen T, Kestelyn P, Collignon J, Goethals M, Belgian Glaucoma S. Screening for glaucoma in a general population with the non-mydriatic fundus camera and the frequency doubling perimeter. European journal of ophthalmology. 2004;14(5):387–93. . 15506600 10.1177/112067210401400505

[18] Tuulonen A, Airaksinen PJ, Montagna A, Nieminen H. Screening for glaucoma with a non-mydriatic fundus camera. Acta ophthalmologica. 1990;68(4):445–9. . 2220362 10.1111/j.1755-3768.1990.tb01674.x

